# Intermediate disturbances are a key driver of long‐term tree demography across old‐growth temperate forests

**DOI:** 10.1002/ece3.8320

**Published:** 2021-11-12

**Authors:** Thomas A. Nagel, Dejan Firm, Andrej Rozman

**Affiliations:** ^1^ Department of Forestry and Renewable Forest Resources Biotechnical Faculty University of Ljubljana Ljubljana Slovenia; ^2^ Scion – New Zealand Forest Research Institute Rotorua New Zealand

**Keywords:** demography, disturbance, forest dynamics, mode of mortality, moderate disturbance, mortality, permanent plots, recruitment, temperate forest

## Abstract

Disentangling the relative influence of background versus disturbance related mortality on forest demography is crucial for understanding long‐term dynamics and predicting the influence of global change on forests. Quantifying the rates and drivers of tree demography requires direct observations of tree populations over multiple decades, yet such studies are rare in old‐growth forest, particularly in the temperate zone of Europe. We use multi‐decade (1980–2020) monitoring of permanent plots, including observations of mode of mortality and disturbance events, to quantify rates and drivers of tree demography across a network of old‐growth remnants in temperate mountain forests of Slovenia. Annual rates of mortality and recruitment varied markedly among sites and over time; census intervals that captured intermediate severity canopy disturbances caused subtle peaks in annual mortality (e.g., >2%/year), while rates of background mortality in non‐disturbed intervals averaged about 1%/year. Roughly half of the trees died from modes of mortality associated with disturbance (i.e., uprooting or snapped‐alive). Results of a Bayesian multilevel model indicate that beech (*Fagus sylvatica*) had a higher likelihood of disturbance related mortality compared to fir (*Abies alba*), which mainly died standing, and there was a notable increase in the odds of disturbance mortality with increasing diameter for all species. Annual recruitment rates were consistently low at sites (<0.5%) that lacked evidence of disturbance, but often exceeded 3% on sites with higher levels of past canopy mortality. Recruitment was dominated by beech on sites with more diffuse background mortality, while the less shade tolerant maple (*Acer pseudoplatanus*) recruited following known disturbance events. Our study highlights the important role of stand‐scale, partial canopy disturbance for long‐term forest demography. These results suggest that subtle climate‐driven changes in the regime of intermediate severity disturbances could have an important influence on future forest dynamics and warrant attention.

## Graphical abstract

We use multi‐decade monitoring of permanent plots to quantify rates and drivers of tree demography across a network of old‐growth remnants in temperate mountain forests. Annual rates of mortality and recruitment varied markedly among sites and over time, and roughly half of all trees died from disturbance related mortality. Our study highlights the important role of stand‐scale, intermediate severity disturbance for long‐term forest demography.

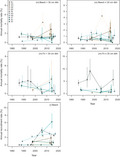

## 1 INTRODUCTION

In the absence of large, severe disturbances, the structure and composition of mesic temperate forests have long been thought to be regulated by relatively continuous formation of small canopy gaps, whereby canopy trees die at a rate of about 1% per year, and tree replacement maintains late successional species (Busing, 2005; Nakashizuka et al., 1992; Runkle, 2000, 2013; Woods, 1984). Given that even small changes in rates of “background” mortality and recruitment can have profound effects on forest dynamics and turnover (van Mantgem et al., 2009; McMahon et al., 2019), there is widespread concern how global change will influence tree demography, with implications for carbon storage, forest biodiversity, and other functions (McDowell et al., 2020). Quantification of the rates and drivers of tree demography are, therefore, needed for robust projections of future forest dynamics (Fisher et al., 2018; McDowell et al., 2018; Senf et al., 2021).

An increasing body of research in temperate forests highlights that on top of background gap dynamics, forest stands periodically experience intermediate severity disturbance events, such as thunderstorms or ice storms (Frankovic et al., 2021; Martin‐Benito et al., 2020; Meigs & Keeton, 2018; Nagel, Mikac, et al., 2017; Pettit et al., 2021). Such events have approximate recurrence intervals of several centuries (Fraver et al., 2009; Frelich & Lorimer, 1991; Nagel et al., 2014), less than the lifespan of most late successional species. Damage patterns are typically characterized by 20–50% canopy removal over a broad range of spatial scales (10s–1000s ha) and are often very “messy,” ranging from scattered gaps to larger patches (i.e., 1000s of m^2^) that retain live trees (Greenberg & McNab, 1998; Hanson & Lorimer, 2007; Nagel & Diaci, 2006; Nagel et al., 2016; Stueve et al., 2011; Woods, 2004). These disturbance events should have a pronounced influence on forest demography, resulting in non‐equilibrial dynamics in temperate old‐growth forests (Mori et al., 2007; Woods et al., 2021), but their role may be difficult to separate from background mortality, particularly when direct, long‐term observations of mortality and recruitment are lacking.

While reconstructive approaches, such as dendroecology (e.g., Firm et al., 2009; Frelich & Lorimer, 1991; Splechtna et al., 2005) or observations of canopy gaps (e.g., Kucbel et al., 2010; Nagel & Svoboda, 2008; Runkle, 1982), can provide insight into past disturbances and forest demography, direct observations of tree populations over sufficient periods of time in old‐growth forests are needed to quantify the rates and drivers of tree demography (e.g., Esquivel‐Muelbert et al., 2020; van Mantgem et al., 2009; Masaki et al., 2021; Tanner et al., 2014; Woods et al., 2021). Long‐term studies are not only needed to capture rare disturbances that influence both mortality and recruitment but also because in the absence of disturbance, variation in annual recruitment rates can be markedly higher than mortality due to the stochasticity of seed production, requiring perhaps decades of observations to capture multiple masting cycles (Clark et al., 1999).

In the temperate zone of Europe, a further challenge to studying tree demography is the long history of land use that has left only scattered remnants of old‐growth forests conditions where natural processes have been the dominant driver of forest dynamics for several centuries. Recent efforts to quantify demographic rates across European forests have relied on data from managed forests (Neumann et al., 2017; Senf et al., 2021), where tree harvesting is the dominant mortality agent, or from forest reserves with a history of past management (Etzold et al., 2019), where stands are still developing toward old‐growth conditions. In a recent study by Woods et al. (2021), multi‐decade data sets from permanent plots in extant old‐growth forest remnants of Europe and North America were used to examine long‐term rates of annual mortality; they found large variation in mortality rates over time, highlighting the importance of rare disturbance events.

We build on the study of Woods et al. (2021) using a network of small old‐growth remnants located across temperate mountain forests of Slovenia, where long‐term observations of tree populations have been carried out in permanent plots since the 1980s. We quantify multi‐decade rates and drivers of tree demography, using data on mode of mortality and disturbance observations to disentangle background and disturbance related mortality. Although tree death is likely a complex process, often with multiple interacting drivers acting in sequence (Franklin et al., 1987; Lugo & Scatena, 1996; Manion, 1981), the mode of mortality is useful for distinguishing between background (i.e., standing dead trees) and abrupt disturbance related mortality (i.e., uprooted or snapped trees) (Esquivel‐Muelbert et al., 2020; McDowell et al., 2018). We predict that periodic partial canopy disturbances are an important cause of tree mortality in these forests, leading to variation in long‐term rates of mortality and recruitment, as well as recruitment opportunities for less shade tolerant tree species.

## 2 STUDY AREA

This research was conducted in 11 old‐growth forest remnants distributed across the Dinaric mountain range and the Southern Limestone Alps in Slovenia (Table 1). All of the sites are part of a strictly protected network of national forest reserves in Slovenia. These forests have structural features characteristic of mesic‐temperate old‐growth forests, including complex stand structure, canopy trees that exceed 80 cm in diameter and 40 m in height, and large amounts of standing and lying deadwood (Nagel et al., 2006, 2015; Nagel, Firm, et al., 2017). Increment cores collected from recently dead canopy trees across the reserves range from c. 200–400 years old (Nagel et al., 2007; Pretzsch et al., 2020). All the sites are located within temperate‐mesic mountain forests, and are primarily dominated by *Fagus sylvatica* (hereafter beech) with *Abies alba* (fir) as a codominant at some sites; *Acer pseudoplatanus* (maple) and *Picea abies* (spruce) are sporadically present at some sites, except for the ZD site, where spruce is dominant (Table 1). Beech and fir are two of the most shade tolerant tree species in European temperate forests, while maple and spruce are considered mid‐tolerant (Leuschner & Ellenberg, 2017). Sites in the Dinaric region are mainly located on upper plateau positions with relatively gentle slopes, whereas sites in the Southern Alps are located on steeper mid‐slope positions. Most of the sites occur on limestone bedrock with well‐drained soils. Precipitation is relatively evenly distributed throughout the year, with an annual average of about 1200 mm at the driest site (DG) to >2300 mm at the wettest sites (ZD, BV); the mean annual temperature ranges from about 5 to 9°C across the sites. None of the permanent plots are located on extreme, inaccessible sites characterized by low productivity. The slope positions and soil conditions are typical of the broader site conditions in temperate mountain forests across the region.

**TABLE 1:** Site and stand characteristics of the old‐growth forest remnants located across mountain regions of Slovenia

Characteristic	BV	DG	GO	Old‐growth site		PE	RR	RG	ST	SU	ZD
				KR	MP						
Remnant size (ha)	8	39	23	74	70	60	52	16	16	19	156
Elevation (m)	1220–1300	580–880	990–1150	840–1170	950–1460	800–910	850–900	860–950	840–940	900–1100	1300–1480
Slope (°)	10–25°	40°	5–10°	0–5°	35–40°	5–20°	0–10°	5–8°	5–10°	35–40°	5–15°
Aspect	W	NW	NE	S	NE	SE	S	NE	SW	W	SE
Basal area (m^2^/ha)	38.5	36.5	41.9	42.5	30.9	37.9	50.2	37.9	45.9	53.8	51.0
Composition (% basal area)	Fasy (89)	Fasy (98)	Fasy (98)	Fasy (90)	Fasy (90)	Fasy (87)	Abal (58)	Fasy (81)	Fasy (72)	Fasy (64)	Piab (67)
	Acps (7)	Acps (2)	Acps (2)	Abal (9)	Acps (5)	Abal (13)	Fasy (41)	Acps (19)	Abal (27)	Abal (29)	Fasy (31)
	Abal (4)			Acps (1)	Piab (5)		Acps (1)		Piab (1)	Piab (6)	Acps (2)

Note

Basal area and composition were calculated from the most recent census. Species include beech (*Fasy*), fir (*Abal*), maple (*Acps*), and spruce (*Piab*). Site names are Bukov vrh (BV), Donačka Gora (DG), Gorjanci (GO), Krokar (KR), Menina Planina (MP), Pečka (PE), Rajhenavski Rog (RR), Ravna Gora (RG), Strmec (ST), Šumik (SU), and Ždrocle (ZD). Site located in the Alps include MP and SU, while all other sites are located in the Dinaric Mountains.

## 3 METHODS

### 3.1 Field measurements

Between the years 1978 and 2012, one to three permanent plots were established within each of 11 old‐growth sites. Overall, there are 19 plots in the network that range in size from 0.2 to 2.1 ha (mean: 0.67 ha), for a total of 12.73 ha (Table 2). All live trees ≥5 cm diameter at breast height (dbh) are labeled with numbered aluminum tags; the original inventories at three sites included stems just below 5 cm dbh, which were retained in our analyses. Following their initial establishment, plots were re‐censused 1–5 times, with census intervals varying from 2 to 31 years (mean: 11 years); plots are currently on a 5‐year re‐census schedule (Table 2). Collectively, the data set contains >7500 tagged trees and 32,000 census observations.

**TABLE 2:** Plot and census characteristics within each old‐growth forest site

	BV	DG	GO	Old‐growth site		PE	RR	RG	ST	SU	ZD
				KR	MP						
Number of plots	2	1	1	2	1	3	3	2	1	1	2
Total plot area (ha)	0.67	0.80	1.00	1.50	0.80	2.90	1.91	1.05	0.50	0.86	0.74
Census years	1985, 2012, 2017	2011, 2016	2012, 2017	1985, 2012, 2017	1992, 2002, 2012, 2017	1980, 1993, 1995, 1998, 2014, 2019	1984, 1994, 2010, 2015, 2020	1983, 2012, 2017	2000, 2012, 2017	1978, 1998, 2012, 2017	1982, 2013, 2018
N trees	419	145	259	561	345	1875	1482	943	205	694	604
Disturbance	Ice‐storm (2014)		Summer storm‐wind (2017)		Summer storm‐wind (2008)	Summer storm‐wind (1983)		Summer storm‐wind (1983)			

At each census, the dbh of each tagged tree was measured, trees that recruited to 5 cm dbh were tagged and recorded, and tree status was noted as alive or dead. Beginning consistently in 2010 (and earlier for some stands), we categorized the mode of mortality for each dead tree in one of the following classes: (1) standing dead; (2) standing dead‐snapped (i.e., a tree that snapped after standing dead, indicated by a relatively smooth‐flat break, decayed crown, and field notes from a previous census); (3) snapped alive (i.e., a tree that snapped while still alive, indicated by a splintered break and intact‐fresh crown); (4) snapped unknown (i.e., a snapped stem that could not be placed into either of the previous categories); (5) uprooted; and (6) snapped or uprooted by another tree (i.e., trees that were clearly crushed during the mortality of other larger individuals). Finally, records of disturbance events (e.g., agent, timing, and damage patterns) that caused damage in the old‐growth sites were maintained throughout the observation period (e.g., Nagel et al., 2006).

### 3.2 Demographic rate calculations

We calculated per capita annual mortality and recruitment rates for each census interval within each site, pooling plots within sites. Rates were calculated for the overall community (all species pooled), for dominant species, and in two size classes for mortality, <30 cm dbh and ≥30 cm dbh (based on the dbh at the beginning of each census). These two size classes reasonably distinguish suppressed understory trees from those with canopy status, but still had sufficient sample sizes within a given census interval in most cases for calculation of rates and confidence intervals. Annual rates were calculated using the definitions proposed by Sheil et al. (1995), Sheil et al. (2000), and summarized in Kohyama et al. (2018). The annual mortality rate, *m*, was quantified as

$$m=1‐{\left(Ns_{T}/N_{0}\right)}^{1/T}$$

where *N*_0_ is the number of trees at the beginning of a census interval, *Ns_T_* is the number of those trees that survived to the end of the census interval, and *T* is the length of the census interval. This measure of mortality assumes a constant probability of mortality during the census interval. Annual recruitment rate, *r*, was quantified as

$$r=1‐{\left(Ns_{T}/N_{T}\right)}^{1/T}$$

where *N_T_* is the number of trees alive at the end of the census interval *T*, and includes both surviving trees that were present in the initial census, and trees that recruited into the population during the census interval. This estimate of recruitment is symmetrical in statistical form with the formulation of annual mortality, *m*, allowing for direct comparison of mortality and recruitment rates (Kohyama et al., 2018; Sheil et al., 2000). It is important to note that variation in census interval length can lead to bias when calculating vital rates from heterogeneous populations, whereby survivorship bias can result in lower rate estimates for longer census intervals (Sheil & May, 1996). Although more recent census intervals across the study sites were fixed at 5 years, many sites had a long census interval following initial plot establishment. Using the approach outlined in Kohyama et al. (2018), we tested for this potential bias using data from the PE site, which includes populations of both beech and fir and large variation in census interval length. Resulting annual rates were nearly identical to the non‐corrected rates, differing by less than one tenth of a percent across species and size classes for each census interval. We therefore continued with analysis of non‐corrected rates calculated using the standard methods described above.

To assess uncertainty in estimates of mortality and recruitment rates, 95% confidence intervals (CIs) were calculated around rates for each census interval; we limit further interpretation of rates to species with sufficient sample sizes (i.e., generally >100 stems for the dominant species (Table S1)). Rather than using the normal approximation to the binomial distribution often employed in previous studies (Condit et al., 1995; Gonzalez‐Akre et al., 2016), we followed the approach of Woods et al. (2021); this approach uses the “Jeffreys interval” based on a beta distribution, which has been shown to provide a more robust and conservative interval estimate compared to the normal approximation (Brown et al., 2001). Using the same approach as Woods et al. (2021), we use these CIs around m and r to indicate ecologically meaningful differences in rates across time at a given site, such as between adjacent intervals that capture disturbance related mortality. However, these estimates are subject to problems associated with multiple comparisons, and census intervals for the same site are not truly independent. To obtain site level estimates of annual mortality, we averaged rates for each site across census intervals, and weighted the estimate for each interval by *N*_0_. We also calculated an overall crude average across sites, weighting each site by the number of census periods. For these estimates, it was not possible to calculate CIs.

### 3.3 Mode of mortality analysis

We used a binomial model with logit link function to evaluate the effect of tree species identity and diameter at breast height on the occurrence of tree mode of mortality associated with disturbances versus background mortality. Only mode of mortality for beech (*N* = 585) and fir (*N* = 184) was used, as they were the only species with sufficient sample sizes and were represented at most of the studied sites. Background mortality comprised standing dead and standing dead‐snapped modes (*N* = 379), while disturbance related mortality included snapped alive and uprooted modes (*N* = 390). A Bayesian analysis framework with Monte Carlo Markov Chain (MCMC) methods was adopted to fit a multilevel logistic regression model with the rstanarm R package (Goodrich et al., 2020). Model estimates are based on four MCMC chains of 2000 iterations each, half of which were discarded as “warm‐up.” We used a weakly informative prior distribution for all the coefficients (i.e., a Student's *t*‐test prior distribution with 7 degrees of freedom, center 0, and a scale of 2.5), as recommended by Gelman et al. (2008). In our random intercept model, we used the “Site” variable as the grouping variable. The dbh variable, originally measured in centimeters, was rescaled to 10‐cm units before model fitting (“dbh10”). Hence, the corresponding regression coefficient represents the effect of the marginal 10‐cm diameter change. We summarized all posterior parameter distributions with regard to their median and 95% credible interval (2.5% and 97.5% quantile of the posterior).

All analyses were performed in the R programming environment (R Core Team, 2020).

## 4 RESULTS

### 4.1 Demography

**FIGURE 1:**
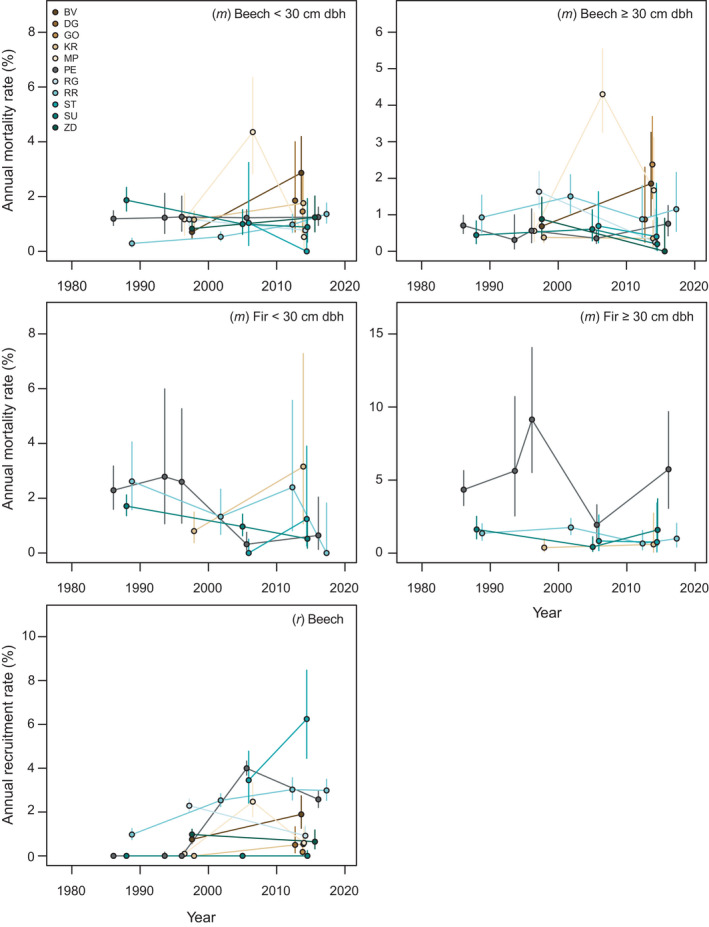
Annual mortality (*m*) and recruitment (*r*) rates for the dominant tree species, fir and beech, in subcanopy and canopy size classes across the old‐growth sites. Colored dots show rates at the mid‐point of each census interval for each site, with 95% confidence intervals

Annual rates of mortality and recruitment varied markedly among sites and over time, as well as by species and size class (Figure 1, Table S2). For some sites, sample sizes within a census interval, particularly for a given species and size class, were often too low to provide a reliable rate with meaningful confidence intervals. However, sites that were affected by moderate severity disturbance events consistently show subtle, but significantly higher mortality rates for certain species or size classes (indicated by non‐overlapping CIs between adjacent census intervals) even when mortality from single events is spread across lengthy census intervals. For example, annual mortality (all species and sizes) was 4.2% (CI: 3.4, 5.1) during the census interval that captured the 2008 thunderstorm event at the MP site and approximately 1% during intervals before (CI: 0.8, 1.6) and after (CI: 0.6, 1.8) this event. Likewise, annual mortality increased from 0.7% (CI: 0.5, 0.9) to 2.1% (CI: 1.5, 2.9) following a 2014 ice storm at the BV site. At the GO site, which was damaged by a summer thunderstorm during the last census, annual mortality was 2.3% (CI: 1.4, 3.6) for canopy‐sized trees. A summer thunderstorm in 1983 caused damage to both the PE and RG sites; the 29‐year census interval that captured this event at RG had an annual mortality rate of 1.5% (CI: 1.1, 2.1) for canopy‐sized trees, which decreased to 0.2% (CI: 0.02, 1.0) in the subsequent census interval. At the PE site, annual mortality of fir has been high throughout most of the study period, reaching 5.4% (CI: 3.5, 7.9) during the 1990s.

Site level averages of annual mortality for all species and sizes pooled ranged from 0.6 to 2.1%, with an overall crude average of 1.3%. If we exclude those sites that captured known past disturbance events (Table 2), the overall average decreases to 1.0%. For beech, the dominant species in the study, site level annual mortality ranged from 0.5 to 2.1%, with an overall crude average of 1.1%. For fir, annual mortality ranged from 0.6 to 3.3% across the sites, with a crude average of 1.8%. Beech was the only species with sufficient sample sizes to allow a comparison between subcanopy and canopy‐sized trees; in 20 of the total 27 census intervals, canopy‐sized stems had lower annual mortality rates, yet confidence intervals indicated these differences were significant in only four cases. Across all sites, the crude average annual mortality for understory and canopy beech trees was 1.2% and 1.0%, respectively.

**FIGURE 2:**
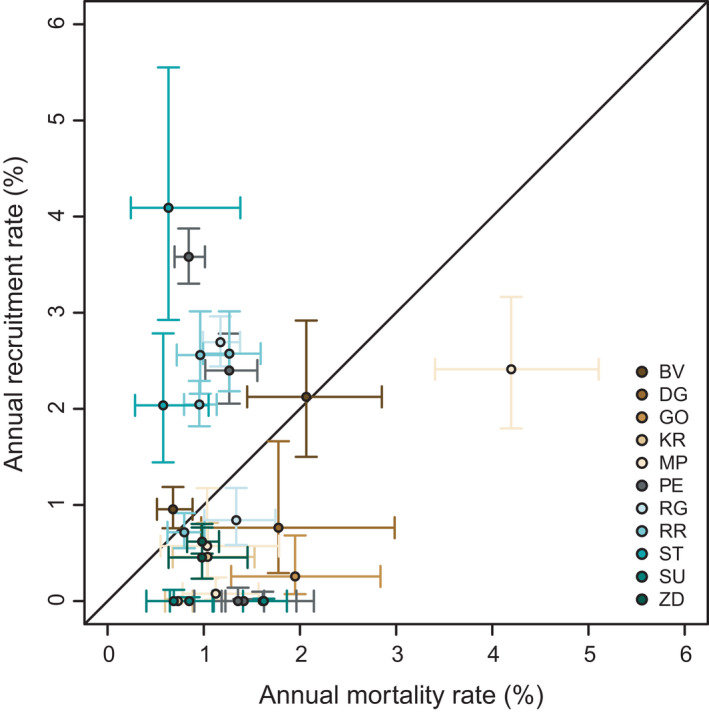
Comparison of community wide (all species and sizes pooled) annual mortality and recruitment rates, with 95% confidence intervals, for all census intervals and sites. The line indicates a 1:1 relationship

There was also notable variability in annual recruitment rates among sites, over time, and by species (Table S3). Based on confidence intervals, recruitment rates significantly outpaced mortality in eight intervals and lagged behind mortality in 10 over all 27 census intervals (Figure 2). While annual recruitment rates reached >3% at some sites (e.g., PE, ST), rates were also very low (e.g., <0.5%) for other sites and census intervals; for example, no stems recruited during the entire 39‐year observation period at the SU site, and only 10 stems recruited at the KR site over 32 years. Moreover, recruitment was overwhelmingly dominated by beech across all sites. Of the 2030 stems that recruited across the entire study period, 89% were beech, 9% were maple, and the remaining stems were a mixture of other species. Annual recruitment rates for beech were often well above 2% within census intervals for a number of sites (e.g., ST, RR, and PE). Although fir was the second most dominant tree in the canopy layer across several of the sites, only four stems at the MP site recruited throughout the entire study. Most of the maple recruitment occurred following the 1983 wind disturbance at the RG site, reaching 3.5% during the census interval that captured this event, and there was also notable maple recruitment at the BV site (3.5%) during the recent interval that captured the ice storm event.

### 4.2 Mode of mortality

**FIGURE 3:**
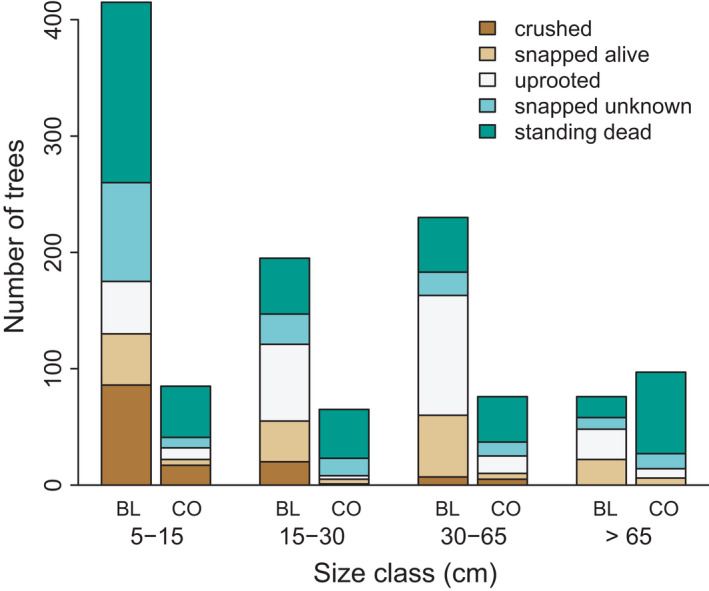
Mode of mortality in four diameter classes for all sites combined. Broadleaf trees (BL) are mainly beech, while conifers (CO) are mainly fir. The standing dead mode includes both standing dead and standing dead‐snapped stems

We recorded the mode of mortality for 1239 stems over the entire study. Excluding those stems that were too decayed to determine a mode, as well as stems that were indirectly killed by other trees, 51% of the stems died from background mortality (i.e., standing dead + standing dead‐snapped) and 49% from disturbance related mortality (i.e., snapped‐alive and uprooted) (Figure 3). There were also clear patterns related to species and tree size. For broadleaf trees, small understory trees (<15 cm dbh) mainly died standing or were killed by other trees, whereas mortality of canopy‐sized (>30 cm dbh) trees was mainly due to disturbance. Conifers showed a contrasting pattern, whereby most stems died standing across all size classes. The numerical and visual convergence diagnostics for the Bayesian multilevel model looked satisfactory (i.e., all chains converged (Rhat < 1.05), and the effective sample sizes were large enough (*N*_eff_/*N* > 0.5). The model results support the patterns outlined above (Table 3); exponentiated coefficients indicate that the odds of disturbance related mortality for beech was 7.92 times that of fir, everything else being equal; and for both species, there was a 17% increase in the odds of disturbance mortality for a 10 cm increase in dbh.

**TABLE 3:** Results of the multilevel logistic model of background (i.e., standing dead + standing dead‐snapped) and disturbance related (i.e., snapped‐alive and uprooted) modes of mortality, including posterior median value, standard deviation, and 95% credible interval of the estimated parameters

	Median	Std. deviation	95% credible interval
Intercept	−1.70	0.46	−2.62 to −0.77
Species | Beech	2.07	0.26	1.56 to 2.60
dbh10	0.16	0.03	0.09 to 0.23
Error terms			
Site (Intercept) 11 levels	1.16		

## 5 DISCUSSION

Our findings show that long‐term tree demography in these old‐growth remnants is driven not only by background mortality but also by discrete disturbance events that cause moderate severity damage that would be difficult to tease apart from background mortality without direct observations. Census intervals that capture these events show subtle, but often significant increases in annual mortality rates compared to non‐disturbed sites, in which annual rates of background mortality averaged about 1% across the entire study period. Moreover, about half of all dead trees across the study were snapped‐alive or uprooted, mortality modes that are associated with disturbance. Finally, consistent with the shade tolerance rankings among the dominant species, there were notable recruitment pulses of maple following disturbance, as well as widespread recruitment of beech in reserves with more diffuse background mortality.

Before we elaborate on these main findings, there are several important caveats that deserve attention and warrant caution when interpreting our results. Following the initial establishment of plots around the 1980s, the long time period before some plots were re‐censused raises several issues. First, any disturbance related mortality is spread across a long interval, yielding rates that are lower than those calculated from shorter census periods. Second, some trees may have recruited to the 5 cm size class and subsequently died prior to being tagged, causing an underestimate of demographic rates; however, across all reserves and census periods during the past decade, we only observed a handful of dead beech trees without tags that were above the recruitment threshold, indicating that such instances of missed recruits were rare. A final caveat includes the confounding influence of indirect anthropogenic factors, namely high deer populations and past air pollution. Populations of ungulates have been high (c. 5–15 deer km^−2^) across much of the study region since 1980s, which is likely the main reason for the recruitment failure of fir and other palatable species in our study (Klopcic et al., 2010; Nagel et al., 2015). High levels of sulfur dioxide emissions in Central Europe during the 1970–1980s have been linked to widespread fir decline and mortality (Elling et al., 2009), which may have contributed to the higher rates of fir mortality at some sites both before and after plot establishment; given that chronic acid deposition results in standing death, we cannot separate such drivers from background mortality. These two chronic stressors complicate any attempt to distinguish between more stable background rates of demography from those associated with abrupt disturbance events. Elevated ungulate populations and air pollution are relatively common drivers of tree demography in other temperate forest regions (Battles & Fahey, 1996; Eschtruth & Battles, 2008), which coupled with other global change drivers (e.g., drought), creates a considerable challenge for studies that try to tease apart drivers of forest demography.

Despite these caveats, the long‐term observation period and regional spatial extent covered by the old‐growth remnants studied here provide a unique opportunity to examine how the disturbance regime in this temperate forest region influences patterns and drivers of demography. Previous work indicates that the disturbance regime in the southeastern Alps and Dinaric Mountain region is complex, characterized by a variety of agents, including wind associated with local thunderstorms, ice‐storms, heavy snow events, and biotic agents (Bebi et al., 2017; Nagel, Mikac, et al., 2017). The study region is among the most thunderstorm prone regions of Europe, with annual frequencies of >40 storms (Nagel, Mikac, et al., 2017; Taszarek et al., 2019). The region also experiences a high frequency of ice storms (Carrière et al., 2000), with severe storms capable of causing damage to forests recurring every few decades (Nagel et al., 2016). When these storm agents reach sufficient intensities, damage to forest canopies is often intermediate in severity and ranges from stands (e.g., a thunderstorm downburst) to larger landscapes (e.g., ice storms), but rarely large‐scale stand replacement (Nagel, Mikac, et al., 2017). The disturbance events captured in the plot network studied here well exemplify such damage patterns. The windthrow events associated with summer thunderstorms at the RG, MP, and GO sites removed 33%, 28%, and 12% of total plot basal area, respectively, resulting in a network of interconnected gaps, ranging from single tree openings to large, multi‐tree gaps up to 600 m^2^ in size at GO and 1900m^2^ at MP (Nagel, personal observations). Likewise, the 2014 ice‐storm at the BV site removed 11% of plot level basal area, and also left many surviving trees with moderate to severe crown damage.

The prevalent mode of mortality associated with these known disturbance events was uprooting (83%), following by bole snapping (17%), and most of these were canopy‐sized trees larger than 50 cm dbh. While a large proportion (40%) of the uprooted and snapped‐alive trees in our study were linked to these known events, the remaining stems with these mortality modes likely occurred during unrecorded lower intensity disturbance events. Clearly, attributing tree death directly to disturbance is not possible, as many of the stems that uprooted or snapped were likely more susceptible due to root and stem decay caused by pathogens (Holzwarth et al., 2013; Worrall et al., 2005), blurring the distinction between background and disturbance related mortality. Standing death was the dominant mode of mortality for fir, particularly for large canopy trees. This mode has often been attributed to air pollution in the study region (Bigler et al., 2004; Diaci et al., 2011; Nagel et al., 2015), yet other agents may have contributed to the high rates of standing dead fir, such as host‐specific bark beetle species or pathogenic fungi (Durand‐Gillmann et al., 2014; Nagel, Mikac, et al., 2017). In other temperate forest regions dominated by conifer species, biotic mortality agents are the prevalent cause of background mortality (Das et al., 2016; Worrall et al., 2005), indicating that further work on their role in background tree mortality is warranted in temperate forests of Europe. Standing death was also common for small pole‐sized trees for both conifers and broadleaf trees, which we suspect is related to both long‐term shading under canopy and density‐dependent competition in gaps; however, these mechanisms were not distinguished in the field. It is also noteworthy that many pole‐sized trees died either directly or indirectly (e.g., crushed by larger trees or snags) from disturbance, which is consistent with mortality patterns observed in a mature broadleaf forest in Central Europe (Holzwarth et al., 2013). On several occasions following heavy snow events in early fall, we observed snapping and uprooting of pole‐sized beech trees growing in gaps, suggesting that these events may be an important mortality agent for small broadleaf trees.

The large variation in recruitment rates observed in our study is at least in part related to known disturbance events or high rates of fir mortality in the past. For example, three reserves (PE, RR, and ST) with high recruitment rates of beech all experienced widespread mortality of fir populations in the past (Nagel et al., 2019). Increased understory light levels caused by diffuse fir mortality, coupled with high browsing pressure on regeneration of palatable species (e.g., fir and maple), likely contributed to the widespread recruitment pulse of beech during the past decade in these reserves (Nagel et al., 2015). The only other species with notable recruitment was maple, particularly at the RG site, which was the only site that captured a relatively severe disturbance sufficiently early in the study period to observe long‐term recruitment patterns in response to disturbance. Maple recruitment outpaced beech within the windthrow area, where pole‐sized maple trees now dominate the canopy layer. There were also sites (SU and KR) that had remarkably low recruitment during the entire observation period; these sites lacked evidence of disturbance and had some of the lowest community wide mortality rates across the entire study. A notable drawback of our data is that we lack information on early demography, from seed production to seedling and sapling survival and recruitment, as well as direct estimates of how deer browsing influences early demography, making it difficult to determine if recruitment dynamics observed in our study are due to disturbance history, browsing pressure or other processes, such as declining fecundity of aging populations (Qiu et al., 2021) or species‐specific pathogens (Szwagrzyk et al., 2015).

Given the scarcity of old‐growth and direct long‐term monitoring of tree populations from permanent plot data in the temperate region of Europe, there are few studies that allow direct comparison with our results. Consistent with our findings, a recent assessment of multi‐decade mortality rates in mesic, temperate old‐growth forests in eastern North America and Europe found that mortality rates were variable over time, with long‐term averages higher than previous published estimates (i.e., generally c. 1%/year), results which were partly attributed to the occurrence of rare disturbance events (Woods et al., 2021). In a long‐term study of mortality rates from permanent plots in mixed forests across Switzerland, Etzold et al. (2019) found an overall average mortality rate of 1.5% during the past 100 years, and a subtle increase in rates over time, which the authors attribute to increasing stand age; while they did find large variation in mortality rates among plots, their study did not examine the influence of disturbance events on mortality rates, and the mean stand age across the study was 130 years, indicating that many stands were recovering from a history of past land use, and as such, may be less susceptible to moderate severity disturbance compared to stands in an old‐growth stage.

Although direct measurements of long‐term demographic rates are scarce, our findings are consistent with recent indirect studies of long‐term forest demography in old‐growth mountain forests of the Carpathians, Dinaric mountains, and Alps. For example, studies that quantified canopy gap metrics typically find that most gaps are small single‐tree openings, but larger multi‐tree gaps measuring several thousand m^2^ in size are frequently encountered (Drösser & von Lüpke, 2005; Kucbel et al., 2010; Nagel & Svoboda, 2008). Likewise, dendroecological reconstructions of disturbance at stand scales often show that intermediate severity disturbances, damaging around 20–40% of the canopy, punctuate periods of background mortality over time scales of several centuries (Firm et al., 2009; Frankovic et al., 2021; Nagel et al., 2014; Splechtna et al., 2005). These studies also document recruitment of less shade tolerant species, such as maple and larch, in larger canopy openings caused by disturbance (Firm et al., 2009; Nagel et al., 2014), underscoring the importance of rare disturbance events for maintaining tree community diversity. Our findings provide further support for a growing body of literature that points out that intermediate severity disturbances have often been overlooked (Hart & Kleinman, 2018; Meigs & Keeton, 2018) and are important for maintaining tree species diversity and stand structural complexity (Fraver et al., 2009; Hanson & Lorimer, 2007; Woods, 2004).

In addition to highlighting the important role of stand‐scale, partial canopy disturbance for long‐term forest demography, our results may have several important implications for understanding how future climate warming will influence forest demography. First, while climate change may result in chronic increases in long‐term rates of background mortality in some regions (e.g., van Mantgem et al., 2009), subtle climate‐driven changes in the regime of intermediate intensity disturbances (e.g., thunderstorms) and their influence on demography warrant attention; recent evidence indicates an increase in annual thunderstorm frequency in some parts of Europe (Taszarek et al., 2019). Second, methodological approaches used to understand forest dynamics under future climate change should not ignore these types of disturbances, particularly simulation models that assign values for demographic rates. Likewise, studies of forest disturbance and mortality that rely on low‐resolution remote sensing (e.g., 0.1 ha) may potentially miss mortality from partial canopy disturbances that damage relatively large areas, but never create gaps larger than a few hundred m^2^ (Senf et al., 2021). Finally, our findings underscore the importance of long observation periods across regional networks of permanent plots for capturing stochastic disturbance events in studies of forest demography (Clark et al., 1999; McMahon et al., 2019).

## CONFLICT OF INTEREST

The authors declare no conflicting interests.

## AUTHOR CONTRIBUTIONS

**Thomas A. Nagel:** Conceptualization (equal); Data curation (lead); Formal analysis (supporting); Funding acquisition (lead); Investigation (equal); Methodology (equal); Project administration (lead); Writing‐original draft (lead). **Dejan Firm:** Conceptualization (equal); Formal analysis (equal); Investigation (supporting); Methodology (equal); Writing‐review & editing (equal). **Andrej Rozman:** Conceptualization (supporting); Formal analysis (equal); Investigation (supporting); Methodology (supporting); Visualization (lead); Writing‐review & editing (equal).

## Supporting information

Appendix S1

Appendix S2

Appendix S3

## Data Availability

Data are publicly available at the Repository of the University of Ljubljana: https://repozitorij.uni‐lj.si/IzpisGradiva.php?id=114849&lang=slv.
