# Supplementary material for: Intermediate disturbances are a key driver of long‐term tree demography across old‐growth temperate forests
Source: Ecol Evol. 2021 Nov 12;11(23):16862–73. doi: 10.1002/ece3.8320 (PMC8668780; doi:10.1002/ece3.8320)
Supplement: Supplementary file 1 — Appendix S1 [file ECE3-11-16862-s002.pdf]

Supporting Information. Nagel, T.A., Firm, D., and Rozman, A. 2021. Intermediate disturbances are a key driver of long-term tree demography across old-growth temperate forests.

Table S1: Sample sizes for calculation of demographic rates for each site and census interval by species and size class (cm). Tables show all species combined, the two dominant species (*Abies alba=Abal; Fagus sylvatica=Fasy*), as well as two less common species (*Acer pseudoplatanus=Acps*; *Picea abies=Piab*) that were present across multiple sites.

N_0 is the number of trees at the beginning of a census interval, and Ns_T is the number of those trees that survived to the end of the census interval;

N_T = number of trees alive at the end of a census interval; N_d = number dead; N_r = number of recruits

BV (Bukov Vrh)

		**1985-2012**					**2012-2017**				
**Species**	**Size**	N_0	N_T	Ns_T	N_d	N_r	N_0	N_T	Ns_T	N_d	N_r
all	all	309	333	257	52	76	333	334	300	33	34
all	<30	188	NA	156	32	NA	211	NA	188	23	NA
all	>=30	121	NA	101	20	NA	122	NA	112	10	NA
Abal	all	4	3	3	1	0	3	3	3	0	0
Abal	<30	0	NA	0	0	NA	0	NA	0	0	NA
Abal	>=30	4	NA	3	1	NA	3	NA	3	0	NA
Acps	all	27	46	24	3	22	46	55	46	0	9
Acps	<30	22	NA	19	3	NA	39	NA	39	0	NA
Acps	>=30	5	NA	5	0	NA	7	NA	7	0	NA
Fasy	all	278	282	230	48	52	282	274	249	33	25
Fasy	<30	166	NA	137	29	NA	170	NA	147	23	NA
Fasy	>=30	112	NA	93	19	NA	112	NA	102	10	NA

DG (Donačka Gora)

		**2011-2016**				
**Species**	**Size**	N_0	N_T	Ns_T	N_d	N_r
all	all	140	133	128	12	5
all	<30	65	NA	57	8	NA
all	>=30	75	NA	71	4	NA
Acps	all	7	6	6	1	0
Acps	<30	3	NA	3	0	NA
Acps	>=30	4	NA	3	1	NA
Fasy	all	126	121	118	8	3
Fasy	<30	56	NA	51	5	NA
Fasy	>=30	70	NA	67	3	NA

GO (Gorjanci)

Species	Size	2012-2017 N_0	N_T	Ns_T	N_d	N_r
all	all	256	235	232	24	3
all	<30	102	NA	95	7	NA
all	>=30	154	NA	137	17	NA
Acps	all	7	7	7	0	0
Acps	<30	3	NA	3	0	NA
Acps	>=30	4	NA	4	0	NA
Fasy	all	249	227	225	24	2
Fasy	<30	99	NA	92	7	NA
Fasy	>=30	150	NA	133	17	NA

KR (Krokar)

		1985-2012					2012-2017				
Species	Size	N_0	N_T	Ns_T	N_d	N_r	N_0	N_T	Ns_T	N_d	N_r
all	all	551	452	452	99	0	452	439	429	23	10
all	<30	282	NA	209	73	NA	188	NA	170	18	NA
all	>=30	269	NA	243	26	NA	264	NA	259	5	NA
Abal	all	72	61	61	11	0	61	56	56	5	0
Abal	<30	41	NA	33	8	NA	27	NA	23	4	NA
Abal	>=30	31	NA	28	3	NA	34	NA	33	1	NA
Acps	all	13	10	10	3	0	10	9	9	1	0
Acps	<30	11	NA	8	3	NA	8	NA	7	1	NA
Acps	>=30	2	NA	2	0	NA	2	NA	2	0	NA
Fasy	all	466	381	381	85	0	381	374	364	17	10
Fasy	<30	230	NA	168	62	NA	153	NA	140	13	NA
Fasy	>=30	236	NA	213	23	NA	228	NA	224	4	NA

MP (Menina planina)

**Species**	**Size**	**1992-2002**					**2002-2012**					**2012-2017**				
		N_0	N_T	Ns_T	N_d	N_r	N_0	N_T	Ns_T	N_d	N_r	N_0	N_T	Ns_T	N_d	N_r
all	all	290	261	259	31	2	261	217	170	91	47	217	212	206	11	6
all	<30	131	NA	110	21	NA	102	NA	65	37	NA	109	NA	107	2	NA
all	>=30	159	NA	149	10	NA	159	NA	105	54	NA	108	NA	99	9	NA
Abal	all	7	5	5	2	0	5	8	4	1	4	8	8	8	0	0
Abal	<30	5	NA	5	0	NA	5	NA	4	1	NA	8	NA	8	0	NA
Abal	>=30	2	NA	0	2	NA	0	NA	0	0	NA	0	NA	0	0	NA
Acps	all	30	24	24	6	0	24	17	17	7	0	17	18	17	0	1
Acps	<30	25	NA	19	6	NA	19	NA	13	6	NA	13	NA	13	0	NA
Acps	>=30	5	NA	5	0	NA	5	NA	4	1	NA	4	NA	4	0	NA
Fasy	all	228	213	211	17	2	213	176	137	76	39	176	171	166	10	5
Fasy	<30	81	NA	72	9	NA	64	NA	41	23	NA	77	NA	75	2	NA
Fasy	>=30	147	NA	139	8	NA	149	NA	96	53	NA	99	NA	91	8	NA
Piab	all	17	16	16	1	0	16	15	11	5	4	15	15	15	0	0
Piab	<30	13	NA	12	1	NA	12	NA	7	5	NA	11	NA	11	0	NA
Piab	>=30	4	NA	4	0	NA	4	NA	4	0	NA	4	NA	4	0	NA

PE (Pečka)

**Species**	**Size**	**1980-1993**					**1993-1995**					**1995-1998**					**1998-2014**					**2014-2019**				
		N_0	N_T	Ns_T	N_d	N_r	N_0	N_T	Ns_T	N_d	N_r	N_0	N_T	Ns_T	N_d	N_r	N_0	N_T	Ns_T	N_d	N_r	N_0	N_T	Ns_T	N_d	N_r
all	all	1117	928	928	189	0	928	903	903	25	0	903	860	860	43	0	860	1346	751	109	595	1346	1426	1263	83	163
all	<30	651	NA	543	108	NA	543	NA	527	16	NA	482	NA	461	21	NA	511	NA	430	81	NA	953	NA	895	58	NA
all	>=30	466	NA	385	81	NA	385	NA	376	9	NA	421	NA	399	22	NA	349	NA	321	28	NA	393	NA	368	25	NA
Abal	all	237	155	155	82	0	155	143	143	12	0	143	121	121	22	0	121	106	106	15	0	106	93	93	13	0
Abal	<30	123	NA	91	32	NA	91	NA	86	5	NA	79	NA	73	6	NA	80	NA	76	4	NA	63	NA	61	2	NA
Abal	>=30	114	NA	64	50	NA	64	NA	57	7	NA	64	NA	48	16	NA	41	NA	30	11	NA	43	NA	32	11	NA
Fasy	all	878	771	771	107	0	771	758	758	13	0	758	737	737	21	0	737	1235	643	94	592	1235	1331	1168	67	163
Fasy	<30	526	NA	450	76	NA	450	NA	439	11	NA	402	NA	387	15	NA	430	NA	353	77	NA	886	NA	832	54	NA
Fasy	>=30	352	NA	321	31	NA	321	NA	319	2	NA	356	NA	350	6	NA	307	NA	290	17	NA	349	NA	336	13	NA

RG (Ravna Gora)

		1983-2012					2012-2017				
**Species**	**Size**	N_0	N_T	Ns_T	N_d	N_r	N_0	N_T	Ns_T	N_d	N_r
all	all	490	768	348	142	420	768	749	718	50	31
all	<30	377	NA	276	101	NA	670	NA	621	49	NA
all	>=30	113	NA	72	41	NA	98	NA	97	1	NA
Acps	all	113	255	90	23	165	255	237	229	26	8
Acps	<30	108	NA	85	23	NA	238	NA	212	26	NA
Acps	>=30	5	NA	5	0	NA	17	NA	17	0	NA
Fasy	all	375	503	257	118	246	503	509	486	17	23
Fasy	<30	267	NA	190	77	NA	422	NA	406	16	NA
Fasy	>=30	108	NA	67	41	NA	81	NA	80	1	NA

RR (Rajhenavski Rog)

		1984-1994					1994-2010					2010-2015					2015-2020				
**Species**	**Size**	N_0	N_T	Ns_T	N_d	N_r	N_0	N_T	Ns_T	N_d	N_r	N_0	N_T	Ns_T	N_d	N_r	N_0	N_T	Ns_T	N_d	N_r
all	all	858	851	792	66	59	851	1016	730	121	286	1016	1102	968	48	134	1102	1178	1034	68	144
all	<30	535	NA	504	31	NA	548	NA	496	52	NA	749	NA	711	38	NA	831	NA	777	54	NA
all	>=30	323	NA	288	35	NA	303	NA	234	69	NA	267	NA	257	10	NA	271	NA	257	14	NA
Abal	all	245	206	206	39	0	206	158	158	48	0	158	150	150	8	0	150	144	144	6	0
Abal	<30	73	NA	56	17	NA	52	NA	42	10	NA	35	NA	31	4	NA	27	NA	27	0	NA
Abal	>=30	172	NA	150	22	NA	154	NA	116	38	NA	123	NA	119	4	NA	123	NA	117	6	NA
Acps	all	12	11	11	1	0	11	9	9	2	0	9	9	9	0	0	9	8	8	1	0
Acps	<30	8	NA	7	1	NA	7	NA	5	2	NA	5	NA	5	0	NA	4	NA	3	1	NA
Acps	>=30	4	NA	4	0	NA	4	NA	4	0	NA	4	NA	4	0	NA	5	NA	5	0	NA
Fasy	all	600	633	574	26	59	633	847	562	71	285	847	941	807	40	134	941	1024	880	61	144
Fasy	<30	454	NA	441	13	NA	489	NA	449	40	NA	708	NA	674	34	NA	799	NA	746	53	NA
Fasy	>=30	146	NA	133	13	NA	144	NA	113	31	NA	139	NA	133	6	NA	142	NA	134	8	NA

ST (Strmec)

Species	Size	2000-2012 N_0	N_T	Ns_T	N_d	N_r	2012-2017 N_0	N_T	Ns_T	N_d	N_r
all	all	134	160	125	9	35	160	191	155	5	36
all	<30	61	NA	58	3	NA	81	NA	79	2	NA
all	>=30	73	NA	67	6	NA	79	NA	76	3	NA
Abal	all	62	60	60	2	0	60	57	57	3	0
Abal	<30	41	NA	41	0	NA	33	NA	31	2	NA
Abal	>=30	21	NA	19	2	NA	27	NA	26	1	NA
Fasy	all	67	93	61	6	32	93	127	92	1	35
Fasy	<30	17	NA	15	2	NA	43	NA	43	0	NA
Fasy	>=30	50	NA	46	4	NA	50	NA	49	1	NA
Piab	all	5	5	4	1	1	5	4	4	1	0
Piab	<30	3	NA	2	1	NA	3	NA	3	0	NA
Piab	>=30	2	NA	2	0	NA	2	NA	1	1	NA

SU (Šumik)

Species	Size	1978-1998 N_0	N_T	Ns_T	N_d	N_r	1998-2012 N_0	N_T	Ns_T	N_d	N_r	2012-2017 N_0	N_T	Ns_T	N_d	N_r
all	all	691	498	498	193	0	498	442	442	56	0	442	427	427	15	0
all	<30	526	NA	360	166	NA	336	NA	292	44	NA	278	NA	269	9	NA
all	>=30	165	NA	138	27	NA	162	NA	150	12	NA	164	NA	158	6	NA
Abal	all	328	233	233	95	0	233	207	207	26	0	207	199	199	8	0
Abal	<30	267	NA	189	78	NA	181	NA	158	23	NA	155	NA	151	4	NA
Abal	>=30	61	NA	44	17	NA	52	NA	49	3	NA	52	NA	48	4	NA
Fasy	all	323	243	243	80	0	243	216	216	27	0	216	210	210	6	0
Fasy	<30	229	NA	157	72	NA	145	NA	126	19	NA	115	NA	110	5	NA
Fasy	>=30	94	NA	86	8	NA	98	NA	90	8	NA	101	NA	100	1	NA
Piab	all	39	21	21	18	0	21	18	18	3	0	18	17	17	1	0
Piab	<30	29	NA	13	16	NA	9	NA	7	2	NA	7	NA	7	0	NA
Piab	>=30	10	NA	8	2	NA	12	NA	11	1	NA	11	NA	10	1	NA

ZD (Ždrocle)

Species	Size	1982-2013					2013-2018				
		N_0	N_T	Ns_T	N_d	N_r	N_0	N_T	Ns_T	N_d	N_r
all	all	512	457	377	135	80	457	445	435	22	10
all	<30	355	NA	247	108	NA	293	NA	272	21	NA
all	>=30	157	NA	130	27	NA	164	NA	163	1	NA
Acps	all	13	10	10	3	0	10	9	9	1	0
Acps	<30	10	NA	7	3	NA	6	NA	5	1	NA
Acps	>=30	3	NA	3	0	NA	4	NA	4	0	NA
Fasy	all	274	286	211	63	75	286	281	272	14	9
Fasy	<30	224	NA	173	51	NA	231	NA	217	14	NA
Fasy	>=30	50	NA	38	12	NA	55	NA	55	0	NA
Piab	all	216	157	152	64	5	157	152	151	6	1
Piab	<30	113	NA	64	49	NA	53	NA	48	5	NA
Piab	>=30	103	NA	88	15	NA	104	NA	103	1	NA
