# Supplementary material for: Intermediate disturbances are a key driver of long‐term tree demography across old‐growth temperate forests
Source: Ecol Evol. 2021 Nov 12;11(23):16862–73. doi: 10.1002/ece3.8320 (PMC8668780; doi:10.1002/ece3.8320)
Supplement: Supplementary file 2 — Appendix S2 [file ECE3-11-16862-s003.pdf]

Supporting Information. Nagel, T.A., Firm, D., and Rozman, A. 2021. Intermediate severity disturbances are a key driver of long-term tree demography across old-growth temperate forests.

Table S2: Annual mortality rate (*m*, expressed as %) with 95% confidence intervals (LCI=lower limt; UCI=upper limit) for each site and census interval by species and size class (cm). Tables show all species combined, the two dominant species *(Abies alba=Abal; Fagus sylvatica=Fasy*), as well as two less common species (*Acer pseudoplatanus=Acps*; *Picea abies=Piab*) that were present across multiple sites.

BV (Bukov Vrh)

		**1985-2012**			**2012-2017**		
**Species**	**Size**	***m***	**LCI**	**UCI**	***m***	**LCI**	**UCI**
all	all	0.68	0.51	0.88	2.07	1.45	2.85
all	<30	0.69	0.48	0.96	2.28	1.49	3.34
all	>=30	0.67	0.42	1.01	1.70	0.87	2.98
Acps	all	0.44	0.12	1.15	0.00	0.00	1.08
Acps	<30	0.54	0.15	1.42	0.00	0.00	1.27
Acps	>=30	0.00	0.00	1.75	0.00	0.00	6.68
Fasy	all	0.70	0.52	0.92	2.46	1.73	3.39
Fasy	<30	0.71	0.48	1.00	2.87	1.87	4.19
Fasy	>=30	0.69	0.43	1.05	1.85	0.95	3.26

DG (Donačka Gora)

		**2011-2016**		
**Species**	**Size**	***m***	**LCI**	**UCI**
all	all	1.78	0.97	2.98
all	<30	2.59	1.23	4.82
all	>=30	1.09	0.37	2.56
Fasy	all	1.30	0.62	2.44
Fasy	<30	1.85	0.71	4.00
Fasy	>=30	0.87	0.25	2.30

GO (Gorjanci)

		2012-2017		
**Species**	**Size**	***m***	**LCI**	**UCI**
all	all	1.95	1.28	2.84
all	<30	1.41	0.63	2.75
all	>=30	2.31	1.40	3.59
Fasy	all	2.01	1.32	2.92
Fasy	<30	1.46	0.65	2.83
Fasy	>=30	2.38	1.44	3.69

KR (Krokar)

		1985-2012			2012-2017		
**Species**	**Size**	***m***	**LCI**	**UCI**	***m***	**LCI**	**UCI**
all	all	0.73	0.60	0.88	1.04	0.68	1.53
all	<30	1.10	0.87	1.38	1.99	1.23	3.06
all	>=30	0.38	0.25	0.54	0.38	0.15	0.83
Abal	all	0.61	0.32	1.05	1.70	0.65	3.67
Abal	<30	0.80	0.38	1.50	3.16	1.07	7.28
Abal	>=30	0.38	0.11	0.99	0.60	0.06	2.73
Fasy	all	0.74	0.60	0.91	0.91	0.55	1.42
Fasy	<30	1.16	0.89	1.47	1.76	0.99	2.90
Fasy	>=30	0.38	0.25	0.56	0.35	0.12	0.84

MP (Menina planina)

Species	Size	1992-2002 *m*	LCI	UCI	2002-2012 *m*	LCI	UCI	2012-2017 *m*	LCI	UCI
all	all	1.12	0.78	1.57	4.20	3.40	5.11	1.04	0.55	1.78
all	<30	1.73	1.11	2.58	4.41	3.16	5.96	0.37	0.08	1.18
all	>=30	0.65	0.33	1.14	4.06	3.09	5.23	1.73	0.86	3.12
Acps	all	2.21	0.92	4.46	3.39	1.51	6.50	0.00	0.00	2.87
Acps	<30	2.71	1.12	5.46	3.72	1.54	7.45	0.00	0.00	3.72
Acps	>=30	0.00	0.00	4.66	2.21	0.23	9.43	0.00	0.00	11.10
Fasy	all	0.77	0.47	1.20	4.32	3.43	5.35	1.16	0.60	2.05
Fasy	<30	1.17	0.58	2.12	4.36	2.84	6.35	0.52	0.11	1.67
Fasy	>=30	0.56	0.26	1.05	4.30	3.26	5.55	1.67	0.79	3.12
Piab	all	0.60	0.06	2.75	3.68	1.39	7.79	0.00	0.00	3.24
Piab	<30	0.80	0.08	3.60	5.25	1.97	11.00	0.00	0.00	4.36
Piab	>=30	0.00	0.00	5.71	0.00	0.00	5.71	0.00	0.00	11.10

PE (Pečka)

Species	Size	1980-1993 *m*	LCI	UCI	1993-1995 *m*	LCI	UCI	1995-1998 *m*	LCI	UCI	1998-2014 *m*	LCI	UCI	2014-2019 *m*	LCI	UCI
all	all	1.42	1.23	1.63	1.36	0.90	1.96	1.61	1.19	2.15	0.84	0.70	1.01	1.26	1.02	1.56
all	<30	1.39	1.14	1.66	1.48	0.89	2.34	1.47	0.94	2.20	1.07	0.86	1.32	1.25	0.96	1.60
all	>=30	1.46	1.17	1.80	1.18	0.58	2.13	1.77	1.15	2.62	0.52	0.35	0.74	1.31	0.87	1.89
Abal	all	3.21	2.57	3.95	3.95	2.17	6.59	5.42	3.52	7.93	0.82	0.48	1.32	2.58	1.45	4.25
Abal	<30	2.29	1.60	3.18	2.79	1.07	5.99	2.60	1.09	5.27	0.32	0.11	0.76	0.64	0.13	2.04
Abal	>=30	4.34	3.26	5.65	5.63	2.55	10.71	9.14	5.52	14.07	1.93	1.02	3.31	5.74	3.07	9.68
Fasy	all	0.99	0.82	1.20	0.85	0.48	1.40	0.93	0.60	1.39	0.85	0.69	1.03	1.11	0.87	1.40
Fasy	<30	1.19	0.95	1.48	1.23	0.65	2.12	1.26	0.74	2.02	1.23	0.97	1.52	1.25	0.95	1.61
Fasy	>=30	0.71	0.49	0.99	0.31	0.06	1.00	0.56	0.24	1.16	0.36	0.21	0.56	0.76	0.42	1.25

RG (Ravna Gora)

		1983-2012			2012-2017		
**Species**	**Size**	***m***	**LCI**	**UCI**	***m***	**LCI**	**UCI**
all	all	1.17	0.99	1.38	1.34	1.01	1.74
all	<30	1.07	0.88	1.29	1.51	1.13	1.97
all	>=30	1.54	1.12	2.07	0.20	0.02	0.95
Acps	all	0.78	0.51	1.15	2.13	1.43	3.05
Acps	<30	0.82	0.53	1.21	2.29	1.53	3.28
Acps	>=30	0.00	0.00	1.63	0.00	0.00	2.87
Fasy	all	1.29	1.08	1.54	0.69	0.41	1.07
Fasy	<30	1.17	0.93	1.45	0.77	0.46	1.22
Fasy	>=30	1.63	1.18	2.19	0.25	0.03	1.15

RR (Rajhenavski Rog)

		1984-1994			1994-2010			2010-2015			2015-2020		
**Species**	**Size**	***m***	**LCI**	**UCI**	***m***	**LCI**	**UCI**	***m***	**LCI**	**UCI**	***m***	**LCI**	**UCI**
all	all	0.80	0.62	1.01	0.95	0.80	1.13	0.96	0.72	1.26	1.27	0.99	1.59
all	<30	0.60	0.41	0.83	0.62	0.47	0.81	1.04	0.75	1.40	1.33	1.01	1.72
all	>=30	1.14	0.81	1.56	1.60	1.26	2.01	0.76	0.39	1.34	1.06	0.61	1.72
Abal	all	1.72	1.24	2.32	1.64	1.23	2.15	1.03	0.49	1.94	0.81	0.34	1.67
Abal	<30	2.62	1.58	4.05	1.33	0.68	2.33	2.40	0.81	5.57	0.00	0.00	1.83
Abal	>=30	1.36	0.88	2.01	1.76	1.26	2.37	0.66	0.22	1.56	1.00	0.42	2.04
Acps	all	0.87	0.09	3.90	1.25	0.25	3.86	0.00	0.00	5.28	2.33	0.25	10.15
Acps	<30	1.33	0.14	5.87	2.08	0.42	6.31	0.00	0.00	9.10	5.59	0.58	22.27
Acps	>=30	0.00	0.00	5.71	0.00	0.00	3.61	0.00	0.00	11.10	0.00	0.00	9.10
Fasy	all	0.44	0.30	0.64	0.74	0.58	0.93	0.96	0.70	1.29	1.33	1.03	1.69
Fasy	<30	0.29	0.16	0.48	0.53	0.39	0.72	0.98	0.69	1.35	1.36	1.03	1.76
Fasy	>=30	0.93	0.52	1.54	1.50	1.04	2.10	0.88	0.37	1.80	1.15	0.55	2.16

ST (Strmec)

Species	Size	2000-2012 *m*	LCI	UCI	2012-2017 *m*	LCI	UCI
all	all	0.58	0.29	1.05	0.63	0.24	1.38
all	<30	0.42	0.12	1.11	0.50	0.10	1.59
all	>=30	0.71	0.30	1.46	0.77	0.22	2.04
Abal	all	0.27	0.06	0.87	1.02	0.29	2.69
Abal	<30	0.00	0.00	0.51	1.24	0.26	3.90
Abal	>=30	0.83	0.17	2.61	0.75	0.08	3.44
Fasy	all	0.78	0.32	1.59	0.22	0.02	1.00
Fasy	<30	1.04	0.21	3.24	0.00	0.00	1.15
Fasy	>=30	0.69	0.23	1.63	0.40	0.04	1.86

SU (Šumik)

Species	Size	1978-1998 *m*	LCI	UCI	1998-2012 *m*	LCI	UCI	2012-2017 *m*	LCI	UCI
all	all	1.62	1.41	1.86	0.85	0.65	1.09	0.69	0.40	1.10
all	<30	1.88	1.61	2.18	1.00	0.73	1.32	0.66	0.32	1.19
all	>=30	0.89	0.60	1.27	0.55	0.30	0.93	0.74	0.31	1.52
Abal	all	1.70	1.38	2.06	0.84	0.56	1.21	0.79	0.37	1.47
Abal	<30	1.71	1.36	2.12	0.97	0.63	1.42	0.52	0.18	1.23
Abal	>=30	1.62	0.98	2.52	0.42	0.12	1.12	1.59	0.54	3.72
Fasy	all	1.41	1.13	1.75	0.84	0.56	1.20	0.56	0.23	1.15
Fasy	<30	1.87	1.47	2.34	1.00	0.62	1.52	0.89	0.34	1.93
Fasy	>=30	0.44	0.21	0.83	0.61	0.29	1.14	0.20	0.02	0.92
Piab	all	3.05	1.86	4.67	1.10	0.31	2.86	1.14	0.12	5.13
Piab	<30	3.93	2.31	6.18	1.78	0.36	5.45	0.00	0.00	6.68
Piab	>=30	1.11	0.23	3.43	0.62	0.07	2.80	1.89	0.20	8.34

ZD (Ždrocle)

Species	Size	1982-2013 *m*	1982-2013 LCI	1982-2013 UCI	2013-2018 *m*	2013-2018 LCI	2013-2018 UCI
all	all	0.98	0.83	1.16	0.98	0.63	1.46
all	<30	1.16	0.96	1.40	1.48	0.94	2.20
all	>=30	0.61	0.41	0.87	0.12	0.01	0.57
Acps	all	0.84	0.23	2.19	2.09	0.22	9.16
Acps	<30	1.14	0.31	2.96	3.58	0.38	15.07
Acps	>=30	0.00	0.00	2.44	0.00	0.00	11.10
Fasy	all	0.84	0.65	1.06	1.00	0.57	1.62
Fasy	<30	0.83	0.62	1.08	1.24	0.71	2.02
Fasy	>=30	0.88	0.48	1.48	0.00	0.00	0.91
Piab	all	1.13	0.87	1.43	0.78	0.32	1.59
Piab	<30	1.82	1.36	2.38	1.96	0.75	4.23
Piab	>=30	0.51	0.30	0.81	0.19	0.02	0.90
