# Supplementary material for: Intermediate disturbances are a key driver of long‐term tree demography across old‐growth temperate forests
Source: Ecol Evol. 2021 Nov 12;11(23):16862–73. doi: 10.1002/ece3.8320 (PMC8668780; doi:10.1002/ece3.8320)
Supplement: Supplementary file 3 — Appendix S3 [file ECE3-11-16862-s001.pdf]

Supporting Information. Nagel, T.A., Firm, D., and Rozman, A. 2021. Intermediate severity disturbances are a key driver of long-term tree demography across old-growth temperate forests.

Table S3: Annual recruitment rate (*r*, expressed as %) with 95% confidence intervals (LCI=lower limt; UCI=upper limit) for each site and census interval.
Tables show all species combined, the two dominant species *(Abies alba=Abal; Fagus sylvatica=Fasy*), as well as two less common species (*Acer pseudoplatanus=Acps*; *Picea abies=Piab*) that were present across multiple sites.

BV (Bukov Vrh)

	**1985-2012**			**2012-2017**		
**Species**	***r***	**LCI**	**UCI**	***r***	**LCI**	**UCI**
all	0.95	0.76	1.19	2.12	1.50	2.92
Acps	2.38	1.52	3.52	3.51	1.74	6.29
Fasy	0.75	0.57	0.98	1.90	1.26	2.74

DG (Donačka Gora)

	**2011-2016**		
**Species**	***r***	**LCI**	**UCI**
all	0.76	0.29	1.66
Acps	0.00	0.00	7.71
Fasy	0.50	0.14	1.33

GO (Gorjanci)

	**2012-2017**		
**Species**	***r***	**LCI**	**UCI**
all	0.26	0.07	0.68
Acps	0.00	0.00	6.68
Fasy	0.18	0.04	0.57

KR (Krokar)

Species	1985-2012			2012-2017		
	r	LCI	UCI	*r*	LCI	UCI
all	0.00	0.00	0.02	0.46	0.24	0.81
Abal	0.00	0.00	0.15	0.00	0.00	0.89
Acps	0.00	0.00	0.90	0.00	0.00	5.28
Fasy	0.00	0.00	0.02	0.54	0.28	0.96

MP (Menina planina)

Species	1992-2002			2002-2012			2012-2017		
	r	LCI	UCI	*r*	LCI	UCI	*r*	LCI	UCI
all	0.08	0.02	0.25	2.41	1.80	3.16	0.57	0.24	1.18
Acps	0.00	0.00	1.03	0.00	0.00	1.45	1.14	0.12	5.13
Fasy	0.09	0.02	0.30	2.47	1.79	3.33	0.59	0.23	1.29
Piab	0.00	0.00	1.53	3.05	1.02	7.01	0.00	0.00	3.24

PE (Pečka)

Species	1980-1993			1993-1995			1995-1998			1998-2014			2014-2019		
	r	LCI	UCI	*r*	LCI	UCI	*r*	LCI	UCI	*r*	LCI	UCI	*r*	LCI	UCI
all	0.00	0.00	0.02	0.00	0.00	0.14	0.00	0.00	0.10	3.58	3.30	3.88	2.40	2.05	2.78
Abal	0.00	0.00	0.12	0.00	0.00	0.87	0.00	0.00	0.69	0.00	0.00	0.15	0.00	0.00	0.54
Fasy	0.00	0.00	0.03	0.00	0.00	0.17	0.00	0.00	0.11	4.00	3.68	4.33	2.58	2.21	2.99

RG (Ravna Gora)

Species	1983-2012			2012-2017		
	r	LCI	UCI	*r*	LCI	UCI
all	2.69	2.44	2.96	0.84	0.58	1.18
Acps	3.53	3.00	4.11	0.68	0.32	1.29
Fasy	2.29	2.01	2.59	0.92	0.60	1.35

RR (Rajhenavski Rog)

	**1984-1994**			**1994-2010**			**2010-2015**			**2015-2020**		
**Species**	***r***	**LCI**	**UCI**	***r***	**LCI**	**UCI**	***r***	**LCI**	**UCI**	***r***	**LCI**	**UCI**
all	0.72	0.55	0.92	2.04	1.82	2.29	2.56	2.16	3.01	2.57	2.18	3.01
Abal	0.00	0.00	0.12	0.00	0.00	0.10	0.00	0.00	0.33	0.00	0.00	0.35
Acps	0.00	0.00	2.21	0.00	0.00	1.68	0.00	0.00	5.28	0.00	0.00	5.90
Fasy	0.97	0.75	1.24	2.53	2.25	2.84	3.03	2.55	3.56	2.99	2.53	3.49

ST (Strmec)

	**2000-2012**			**2012-2017**		
**Species**	***r***	**LCI**	**UCI**	***r***	**LCI**	**UCI**
all	2.04	1.44	2.79	4.09	2.92	5.55
Abal	0.00	0.00	0.35	0.00	0.00	0.87
Fasy	3.45	2.41	4.78	6.24	4.45	8.47

SU (Šumik)

	**1978-1998**			**1998-2012**			**2012-2017**		
**Species**	***r***	**LCI**	**UCI**	***r***	**LCI**	**UCI**	***r***	**LCI**	**UCI**
all	0.00	0.00	0.03	0.00	0.00	0.04	0.00	0.00	0.12
Abal	0.00	0.00	0.05	0.00	0.00	0.09	0.00	0.00	0.25
Fasy	0.00	0.00	0.05	0.00	0.00	0.08	0.00	0.00	0.24
Piab	0.00	0.00	0.59	0.00	0.00	0.98	0.00	0.00	2.87

ZD (Ždrocle)

	**1982-2013**			**2013-2018**		
**Species**	***r***	**LCI**	**UCI**	***r***	**LCI**	**UCI**
all	0.62	0.49	0.77	0.45	0.23	0.80
Acps	0.00	0.00	0.79	0.00	0.00	5.28
Fasy	0.98	0.77	1.22	0.65	0.32	1.18
Piab	0.10	0.04	0.23	0.13	0.01	0.61
